# Author Correction: A GDSL lipase-like from *Ipomoea batatas* catalyzes efficient production of 3,5-diCQA when expressed in *Pichia pastoris*

**DOI:** 10.1038/s42003-020-01488-x

**Published:** 2020-12-02

**Authors:** Sissi Miguel, Guillaume Legrand, Léonor Duriot, Marianne Delporte, Barbara Menin, Cindy Michel, Alexandre Olry, Gabrielle Chataigné, Aleksander Salwinski, Joakim Bygdell, Dominique Vercaigne, Gunnar Wingsle, Jean Louis Hilbert, Frédéric Bourgaud, Alain Hehn, David Gagneul

**Affiliations:** 1 Plant Advanced Technologies, Vandoeuvre-lès-Nancy, France; 2 UMR Transfrontalière BioEcoAgro N° 1158, Univ. Lille, INRAE, Univ. Liège, UPJV, ISA, Univ. Artois, Univ. Littoral Côte d’Opale, ICV – Institut Charles Viollette, 59000 Lille, France; 3 Université de Lorraine-INRAE, LAE, 54000 Nancy, France; 4 Chemistry Department, Umeå University, 90183 Umeå, Sweden; 5 Umeå Plant Science Centre, Department of Forest Genetics and Plant Physiology, Swedish University of Agricultural Sciences, 90183 Umeå, Sweden

**Keywords:** Metabolic engineering, Secondary metabolism

Correction to: *Communications Biology* 10.1038/s42003-020-01387-1, published online 13 November 2020.

In the original published version of the Article, co-author Frédéric Bourgaud was inadvertently omitted from the list of Corresponding Authors. The error has been corrected in the PDF and HTML versions of the paper.

