# Supplementary material for: A GDSL lipase-like from Ipomoea batatas catalyzes efficient production of 3,5-diCQA when expressed in Pichia pastoris
Source: Commun Biol. 2020 Nov 13;3:673. doi: 10.1038/s42003-020-01387-1 (PMC7666189; doi:10.1038/s42003-020-01387-1)
Supplement: Supplementary file 1 — Supplementary Information [file 42003_2020_1387_MOESM1_ESM.pdf]

```
IbHCT              MKISVKESTM VRPAEETPRI RLWNSNVDLV VPNFHTPSVY FYRPNGADDF
IbHCT BAJ14794     MKISVKESTM VRPAEETPRI RLWNSNVDLV VPNFHTPSVY FYRPNGADDF
CcHCT AAZ80046     MKIEVKESTM VRPAQETPRR NLWNSNVDLV VPNFHTPSVY FYRPTGSSNF

IbHCT_             FESKALKDGL SRALVEFYPM AGRLTRDEDG RIEIDCNGAG VLLVEAESDG
IbHCT BAJ14794     FESKALKDGL SRALVEFYPM AGRLTRDEDG RIEIDCNGAG VLLVEAESDG
CcHCT AAZ80046     FDAKVLKDAL SRALVEFYPM AGRLKRDEDG RIEIECNGEG VLFVEAESDG

IbHCT              FVDDFGDFAP TLELRQLIPT VDYSQGIESY PLLVLQVTHF KCGGVSLGVG
IbHCT BAJ14794     FVDDFGDFAP TLELRQLIPT VDYSQGIESY PLLVLQVTHF KCGGVSLGVG
CcHCT AAZ80046     VVDDFGDFAP TLELRRLIPA VDYSQGISSY ALLVLQVTYF KCGGVSLGVG

IbHCT              MQHHAADGAS GLHFINTWSD MARGLDLTVP PFIDRTLLRA REPPQPQFQH
IbHCT BAJ14794     MQHHAADGAS GLHFINTWSD MARGLDLTVP PFIDRTLLRA REPPQPQFQH
CcHCT AAZ80046     MQHHAADGFS GLHFINSWSD MARGLDVTLP PFIDRTLLRA RDPPQPQFQH

IbHCT              VEYQPPPALK ASQP-----Q NATSDTAVSI FKLSRDQISA LKAKSKEDGN
IbHCT BAJ14794     VEYQPPPALK APQP-----Q NVTSDTAVSI FKLSRDQISA LKAKSKEDGN
CcHCT AAZ80046     IEYQPPPTLK VSPQ-TAK-S DSVPETAVSI FKLTREQISA LKAKPKEDGN

IbHCT              TVAYSSYEML AGHVWRCACR ARGLTEDQET KLYIATDGRS RLRPSLPAGY
IbHCT BAJ14794     TVAYSSYEML AGHVWRCACR ARGLTEDQET KLYIATDGRS RLRPSLPTGY
CcHCT AAZ80046     TISYSSYEML AGHVWRCACK ARGLEVDQGT KLYIATDGRA RLRPSLPPGY

IbHCT              FGNVIFTATP IAVSGDLQSK PVWFTASKIH GALGRMDNDY LRSALDYLEF
IbHCT BAJ14794     FGNVIFTATP IAVAGDLQSK PVWFAASKIH GALGRMDNDY LRSALDYLEL
CcHCT AAZ80046     FGNVIFTATP IAIAGDLEFK PVWYAASKIH DALARMDNDY LRSALDYLEL

IbHCT              QPDLKALVRG AHTFRCPNLG ITSWVRLPIH DADFGWGRPI FMGPGGIAYE
IbHCT BAJ14794     QPDLKALVRG AHTFRCPNLG ITSWVRLPIH DADFGWGRPI FMGPGGIAYE
CcHCT AAZ80046     QPDLKALVRG AHTFKCPNLG ITSWVRLPIH DADFGWGRPI FMGPGGIAYE

IbHCT_             GLSFMLPSPE NDGSLSIAIS LQEEHMKLFK EFLYDI
IbHCT BAJ14794     GLSFMLPSPE NDGSLSIAIS LQEEHMKLFK EFLYDI
CcHCT AAZ80046     GLSFILPSPT NDGSMSVAIS LQGEHMKLFQ SFLYDI
```

Supplementary Figure 1: Alignment of the amino acid sequence of IbHCT with that of a previously identified HCT from *I. batatas* (AB047805) and that of an HCT from *Cyanara cardunculus* (AAZ80046).
The amino acids (AAs) highlighted in green are different between sequences from *I. batatas* and *C. cardunculus*. The AAs highlighted in yellow are different between the IbHCT cloned in this work and the sequences available in the databank. Conserved motifs are in black boxes. AAs predicted to interact with both substrates are highlighted by a blue scar. AAs predicted to interact with hydroxycinnamic acid derivatives are highlighted by a red dot. AAs predicted to interact with CoA are highlighted by an orange dot.

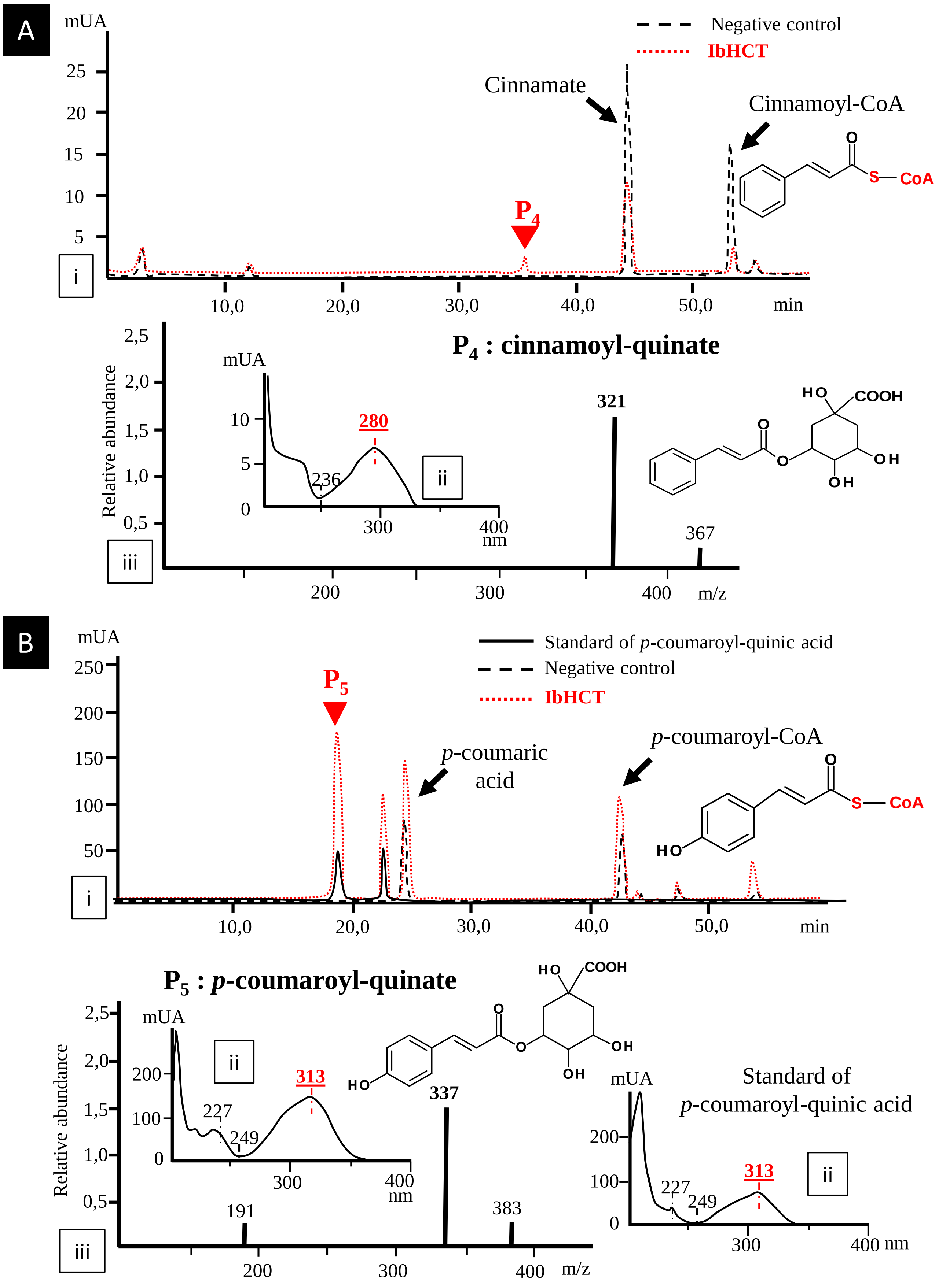

Supplementary Figure 2: Synthesis of cinnamoyl-quinate (A) and *p*-coumaroyl-quinate (B) by incubation of cinnamoyl- and *p*-coumaroyl-CoA, respectively, with quinic acid in the presence of IbHCT for 1 h at 30°C in pH 7-established conditions i) HPLC profile of the enzymatic reaction (330 nm), ii) UV spectrum and iii) MS analysis.

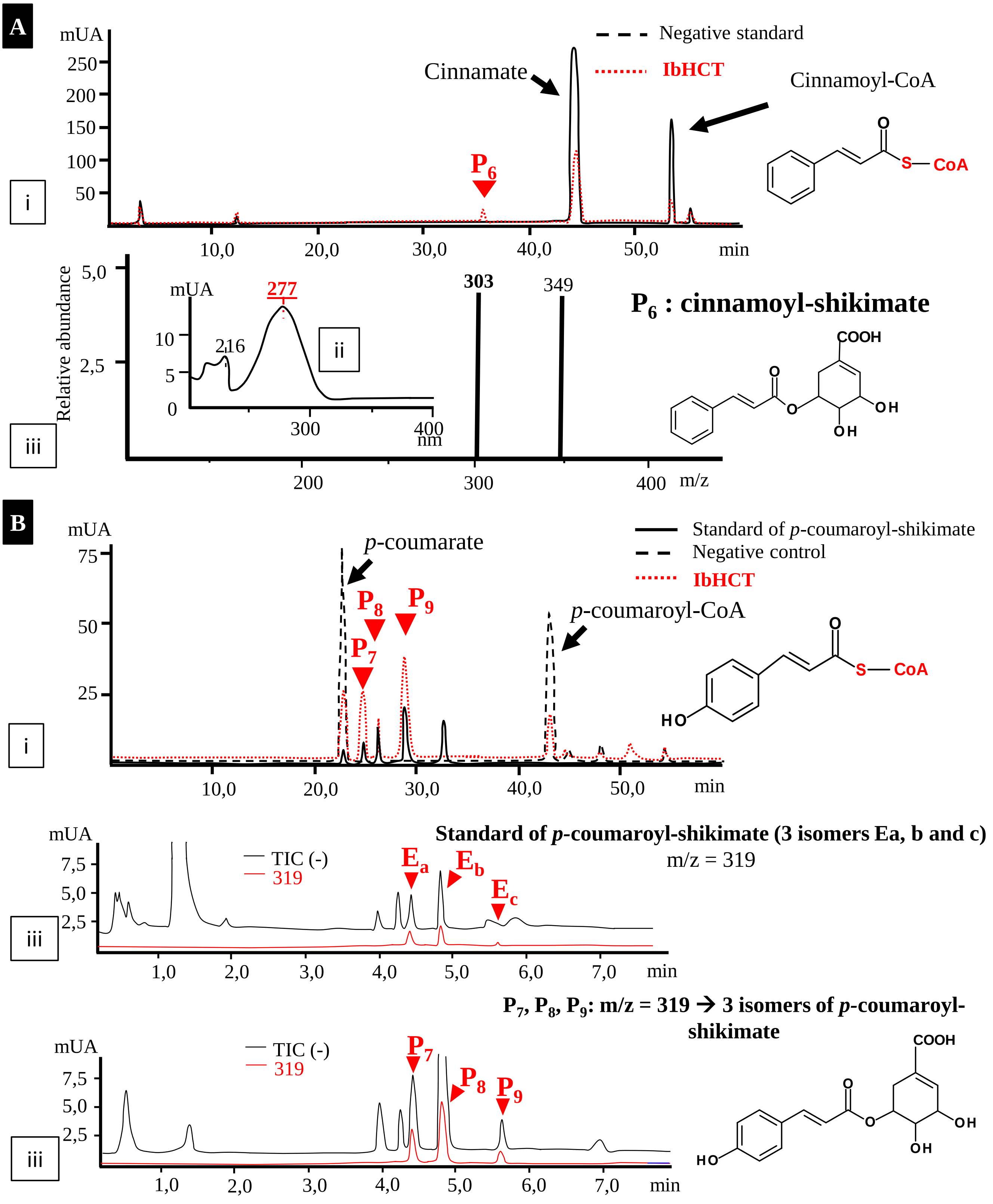
A
mUA
250
200
150
100
50
10,0
20,0
30,0
40,0
50,0
min
i
Negative standard
IbHCT
Cinnamate
Cinnamoyl-CoA
P6
O
S— CoA
Relative abundance
5,0
2,5
iii
mUA
277
10
216
5
0
300
400
nm
ii
303
349
200
300
400
m/z
P6 : cinnamoyl-shikimate
COOH
O
O
OH
OH
B
mUA
75
50
25
i
10,0
20,0
30,0
40,0
50,0
min
p-coumarate
P8
P9
P7
p-coumaroyl-CoA
Standard of p-coumaroyl-shikimate
Negative control
IbHCT
O
S— CoA
HO
mUA
7,5
5,0
2,5
iii
TIC (-)
319
Ea
Eb
Ec
1,0
2,0
3,0
4,0
5,0
6,0
7,0
min
Standard of p-coumaroyl-shikimate (3 isomers Ea, b and c)
m/z = 319
P7, P8, P9: m/z = 319 → 3 isomers of p-coumaroyl-shikimate
mUA
7,5
5,0
2,5
iii
TIC (-)
319
P7
P8
P9
1,0
2,0
3,0
4,0
5,0
6,0
7,0
min
COOH
O
O
OH
OH
HO

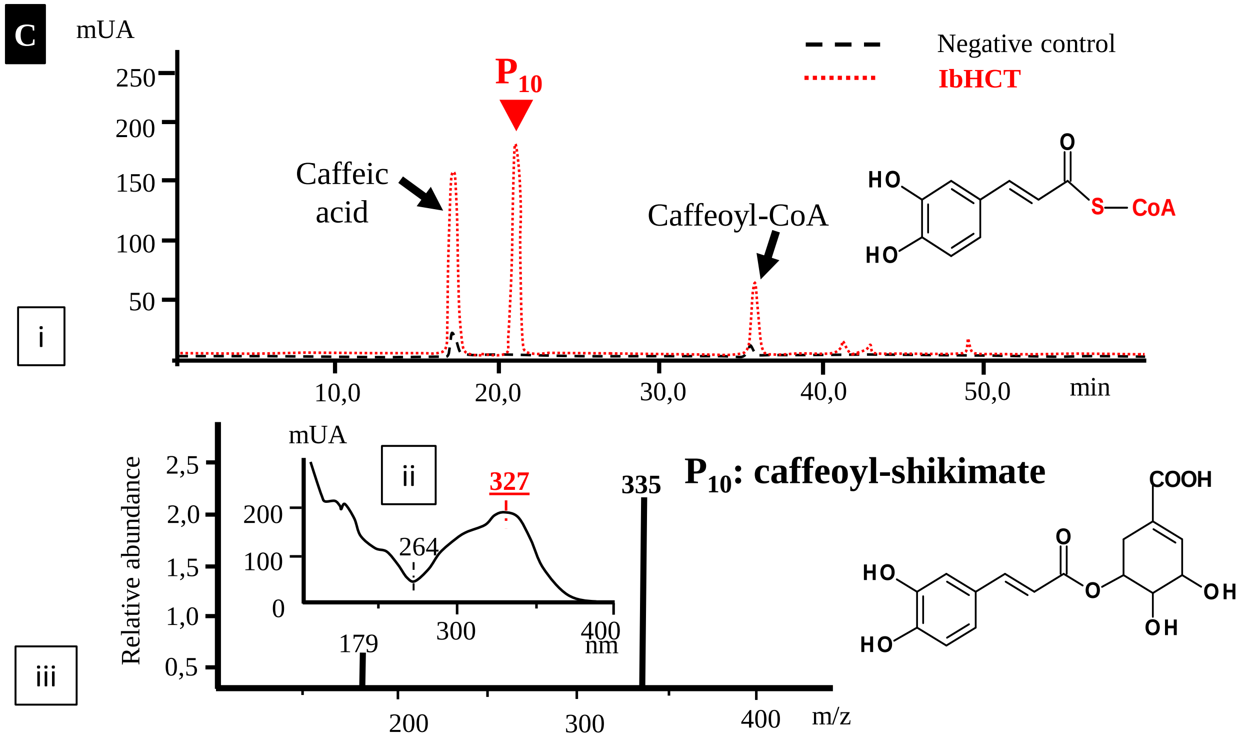

Supplementary Figure 3: Synthesis of cinnamoyl-shikimate (A), *p*-coumaroyl-shikimate (B) and caffeoyl-shikimate (C) by incubation of cinnamoyl-, *p*-coumaroyl- and caffeoyl-CoA, respectively, with shikimic acid in the presence of IbHCT for 1 h at 30°C at 30°C in pH 7-established conditions. i) HPLC analysis (330 nm) ii) UV spectrum and iii) MS analysis.

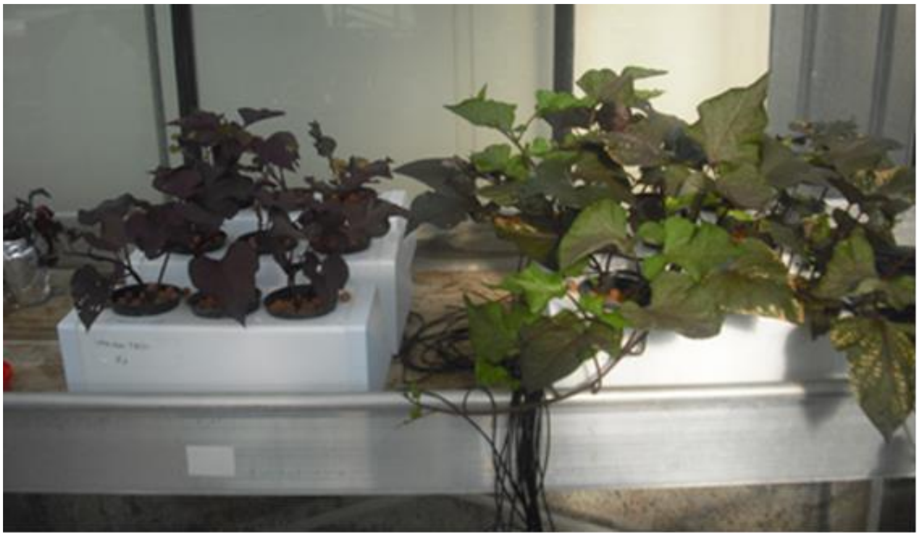

Supplementary Figure 4: Sweet potato cultured in hydroponic conditions. Left in nitrogen-free medium, right in the presence of NPK (15/10/30).

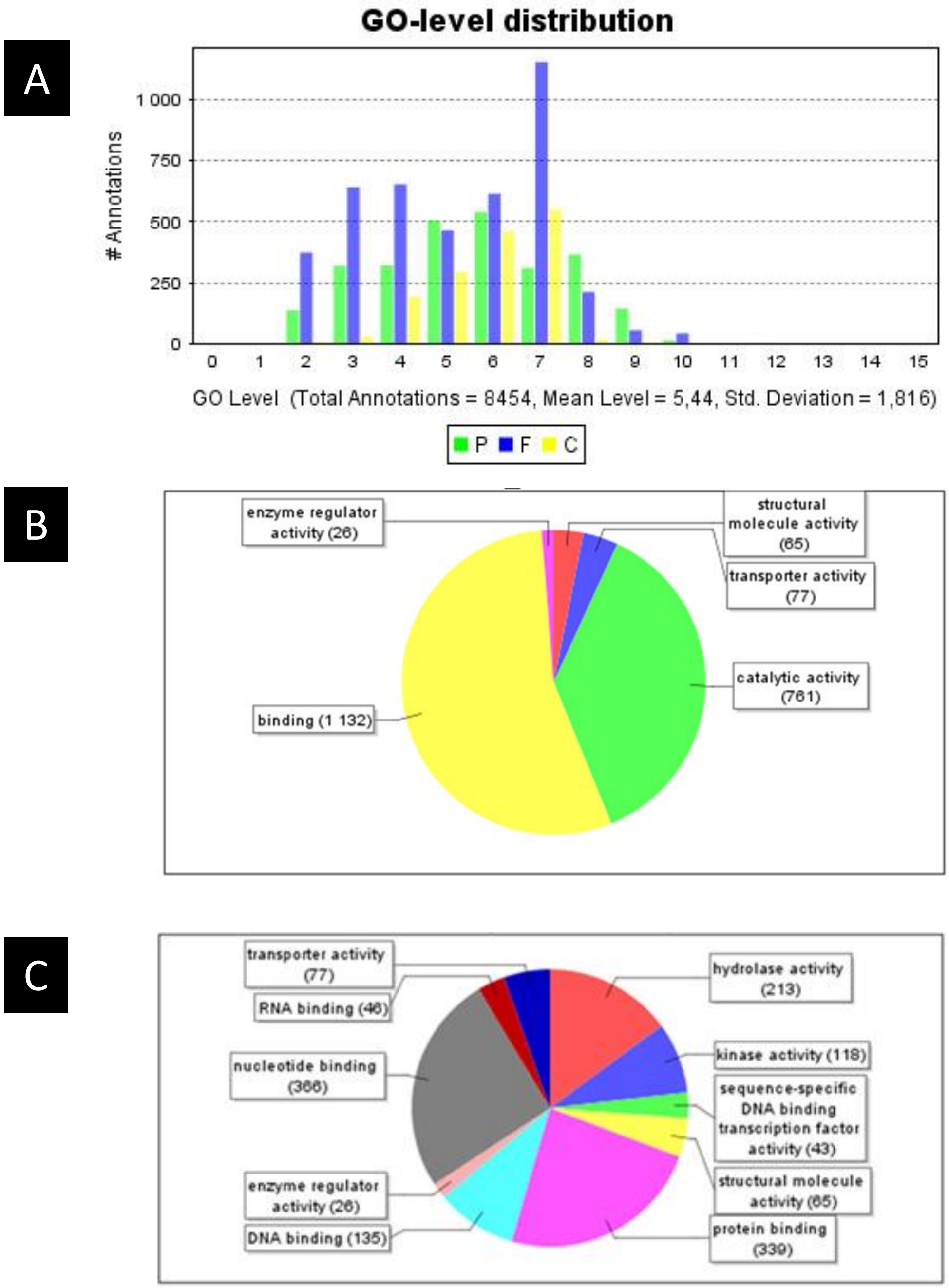

Supplementary Figure 5: Analysis of the *I. batatas* RNA-seq Library.
A) Each sequence is associated with 4 up to 7 annotations GO (P=biological process, F= molecular function, C= cellular compounds) B) Detailed analysis of the molecular functions associated with the contigs. C) Analysis of the predicted catalytic functions.

Supplementary Figure 6: Phylogenetic tree of amino acid sequences of acyltransferases of the BAHD family (adapted from D'Auria et al. [58]). The phylogenetic reconstruction was generated by the neighbour-joining method and displayed using the MEGAX program with bootstrap values of 1000 replicates. Bootstrap values (%) are shown at nodes. HCT is highlighted in orange, and HQT is highlighted in green. Sequences used for alignments: *AsHHT1: BAC78633; AT1: AET80688; AtCER2: AAM64817; AtHCT: NP_199704.1; CbBEAT: AAN48360; CbHCT2: CBI83579; CbRAS: A0PDV5; CcHCT: ABO47805; CcHQT: ABO77957; CyHQT: ABK79689; CsHCT: NP_001295843.1; CsHQT: ACF3707957; DcHCBT2: O23917; Dv3MAT: AAO12206; EcHQT: AFF19203; EcoATA: NP_312354.1; Gt5AT: BAA74428; HvACT: AAO73071; IbHCT: BAJ14794; IbHCBT: BAA87043; IbHQT: BAJ14795; LaAAT1: ABI48360; LjHQT: ACZ52698; MtHCT: XP_013454529; NtHCBT: AAN09798; NtHCT: CAD47830; OsHCT4: NP_001057003; PgHCT: AEM63675; PvHCT1: AFY17066; RsVs: CAD89104; SbHCT: EES05411; NtHQT: CAE46932; SlHQT: NP_001234850; SpAAT1: NP_001310384; StHQT: ABA46756; TpHCT1: ACI16630.*

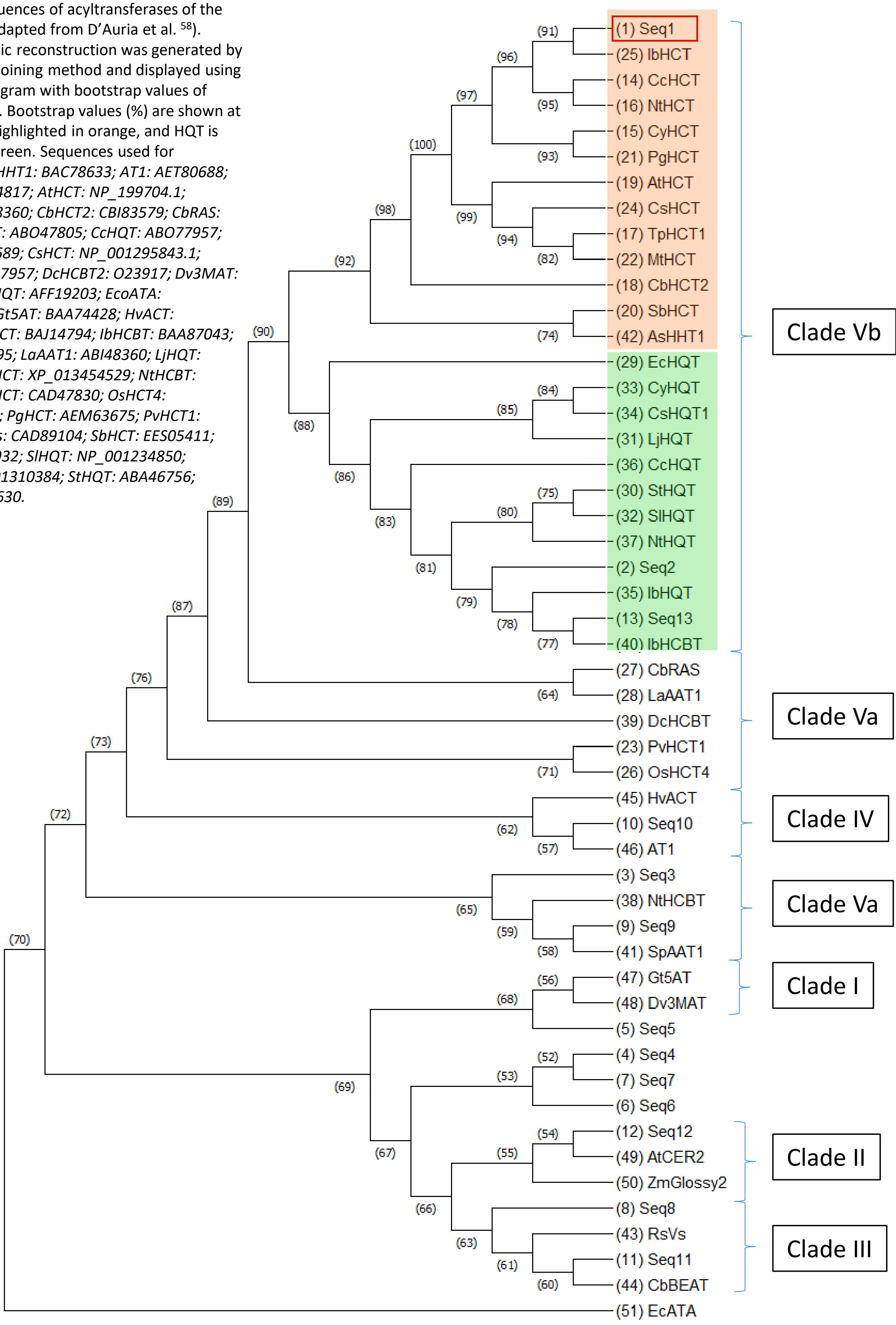

A) Coding Sequence

ATGGCCGCCGCAGCTCCTTCTTCCTCGTTGCTTGGTGTCCTGATGCTGTTAGCTTCCATTGGCTACGCCGCCGCT
TGCTATTCGGCCGTTTTCGGCTTCGGCGATTCCCTCACGGATGCCGGAAACCTAATCCGCCTTGAGCCTGACGGC
ACCGTCCCCCACATGTACTTCCCGCCCTACGGCGAAACCTACTTCGACAAGCCCACCGGACGCTGCTCCGACGGC
CGCCTCATCGTCGATTTAATCGCTCAGCATTACGGGCTTCCGCTTCCGCCGCCTTCGATCCCGGCCAGTTTGGAA
GGCGACAAAAACCGCCTCCGTGCCGGAGTCAACTTCGCGGTGGTCGGATCACGTGCGCTCGACGCGCAATTCTAC
GAACAGAGAGACATTTTCGACACTGTCACCAACGTTTCCATGACAGATCAGTTGAATTGGTTCAAGGAATTGCTC
CCATCTCTGTGCTCATCACCTCACGATTGCAAGGAGCTATTGGATGGGTCATTGTTTGTACTAGGAGAATTTGGA
GGCAACGATTACACGCACTCGCTTCTATCCGGAAAAGGTATCAATGTCATCCGACCGTTCATTCCAATCGTCACC
CGCGCAATTGCTCAGGCTGTCCATGAATTAGTTGAGCTAGGTGCGAGGACAGTAATGATCCCCAGCGTGTTACCA
CTTGGATGTGCAGCCTCGTACTTGACATACTACCCAAGTCCCAACCCAGATGACTACGATGAACTAGGTTGTCTC
ATTTGGGTTAACGAAATGGCTTCTTACCACAACCAACTCCTCCAGCAACAACTCGCCCACGTAAGAGAACTTCAT
CCCTATGCCAATATCGTCTTCGCCGATATTTACAATGCCGCCATGGAGGTTTACCAGGGTCCCGATACTTATGGG
TTTTCCGGGGGAGCTTTGAGAGCATGTTGTGGCGGCGGAGGTCCGTACAACTTCAACACCTCCGCACAATGCGGT
AACGAGGGAGCAACCGCCTGTGACAACCCTTCCTCGTACGTTAACTGGGACGGTTATCACTTAACGGAGGCTGCC
TATCAATGTATTACCACCGGTTTGTTAGAGGGTCCGTATACCTATCCTCGTATGAAAAATTTGTGTCGCTTTGAC
TCTCGCAAAACATCTCGAGTTGCCCAAATTTAA

B) Predicted protein

MAAAAPSSSLLGVLMLLASIGYAAACYSAVFGFGDSLTDAGNLIRLEPDGTVPHMYFPPYGETYFDKPTGRCSDG
RLIVDLIAQHYGLPLPPPSIPASLEGDKNRLRAGVNFAVVGSRALDAQFYEQRDIFDTVTNVSMTDQLNWFKELL
PSLCSSPHDCKELLDGSLFVLGEFGGNDYTHSLLSGKGINVIRPFIPIVTRAIAQAVHELVELGARTVMIPSVLP
LGCAASYLTYYPSPNPDDYDELGCLIWVNEMASYHNQLLQQQLAHVRELHPYANIVFADIYNAAMEVYQGPDTYG
FSGGALRACCGGGGPYNFNTSAQCGNEGATACDNPSSYVNWDGYHLTEAAYQCITTGLLEGPYTYPRMKNLCRFD
SRKTSRVAQI*

Supplementary Figure 7: Nucleotide and predicted protein sequences of *IbICS* deduced from the *I. batatas* RNA-seq library. A) Coding sequence deduced from the RNA-seq library, B) Deduced amino acid sequence.

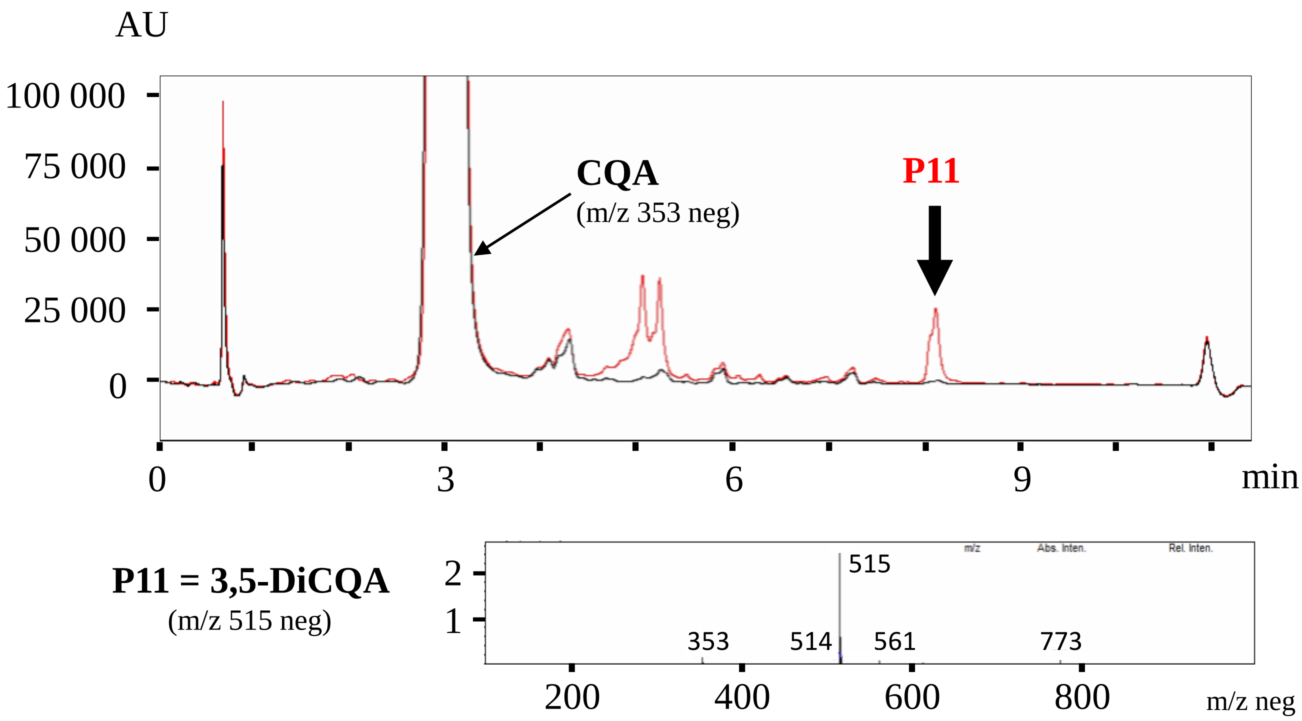

Supplementary Figure 8: Incubation performed with crude protein extract from *N. benthamiana* leaves agroinfiltrated by empty vector (black) or vector harbouring the *IbICS* gene. Incubations were performed in sodium phosphate buffer with a pH set at 6.5 in the presence of 50 mM 5-CQA for 22 h at 28°C. Analysis was performed at 330 nm. 5-CQA and 3,5-DiCQA were identified by MS in comparison to commercial standards.

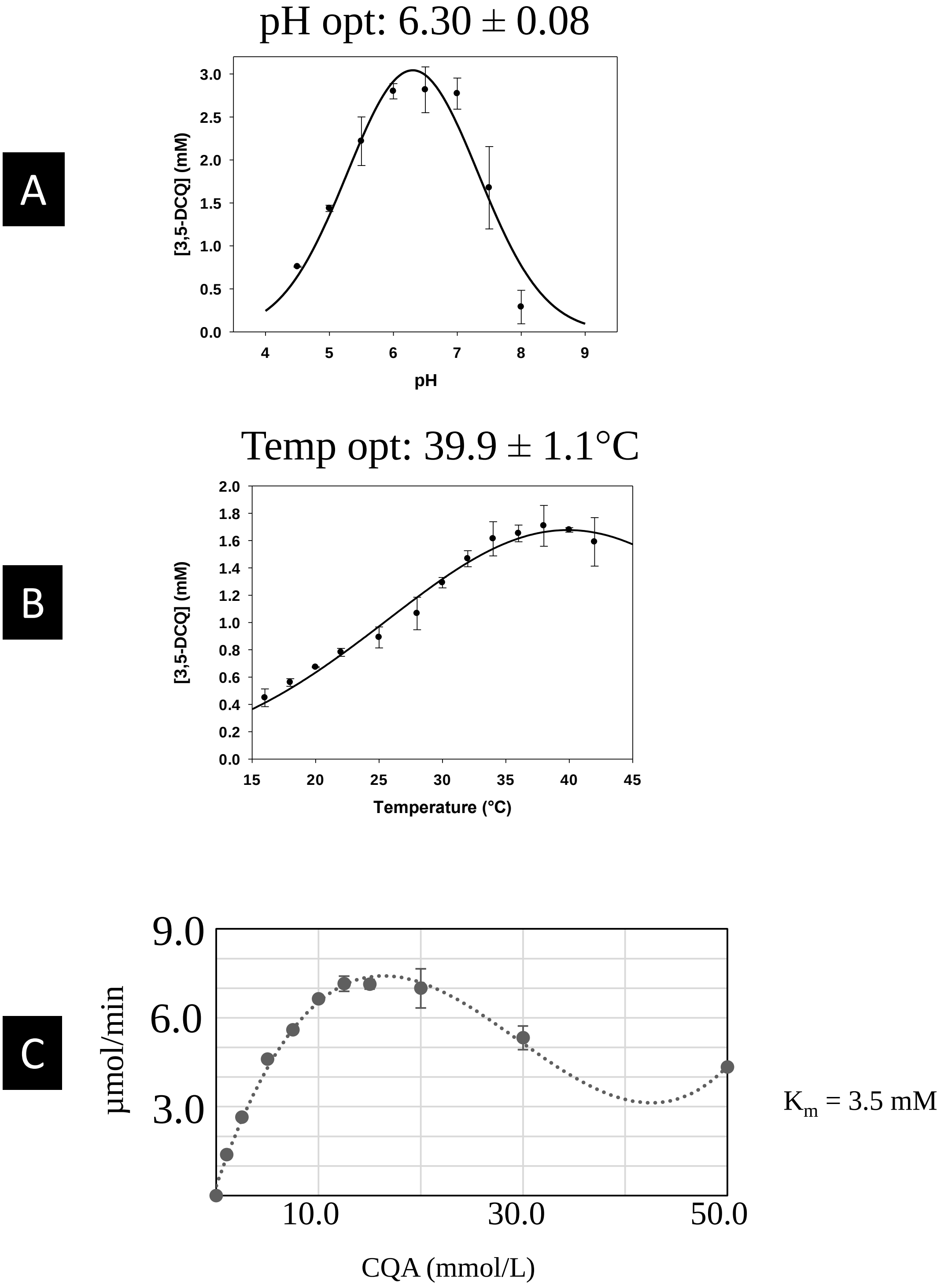

Supplementary Figure 9: Biochemical characterization of IbICS.
The IbICS-HIS enzyme was produced in *N. benthamiana* leaves and subsequently purified. A) Optimal pH, B) Optimal temperature, C) Kinetic parameters. To determine Km and Vm, incubations were performed at 36°C for 30 min at pH 6.5.

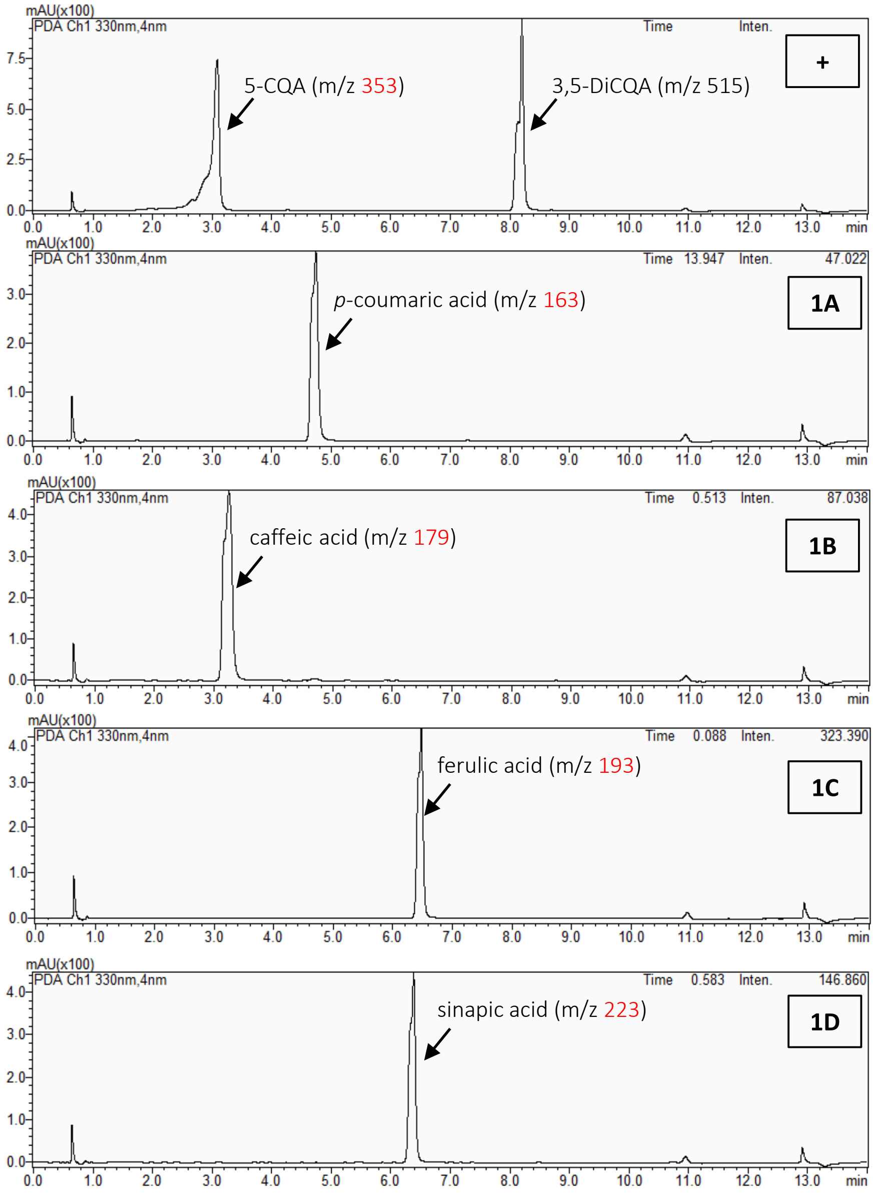
mAU(x100)
PDA Ch1 330nm,4nm
Time
Inten.
+
5-CQA (m/z 353)
3,5-DiCQA (m/z 515)
PDA Ch1 330nm,4nm
Time 13.947 Inten. 47.022
1A
p-coumaric acid (m/z 163)
PDA Ch1 330nm,4nm
Time 0.513 Inten. 87.038
1B
caffeic acid (m/z 179)
PDA Ch1 330nm,4nm
Time 0.088 Inten. 323.390
1C
ferulic acid (m/z 193)
PDA Ch1 330nm,4nm
Time 0.583 Inten. 146.860
1D
sinapic acid (m/z 223)
min

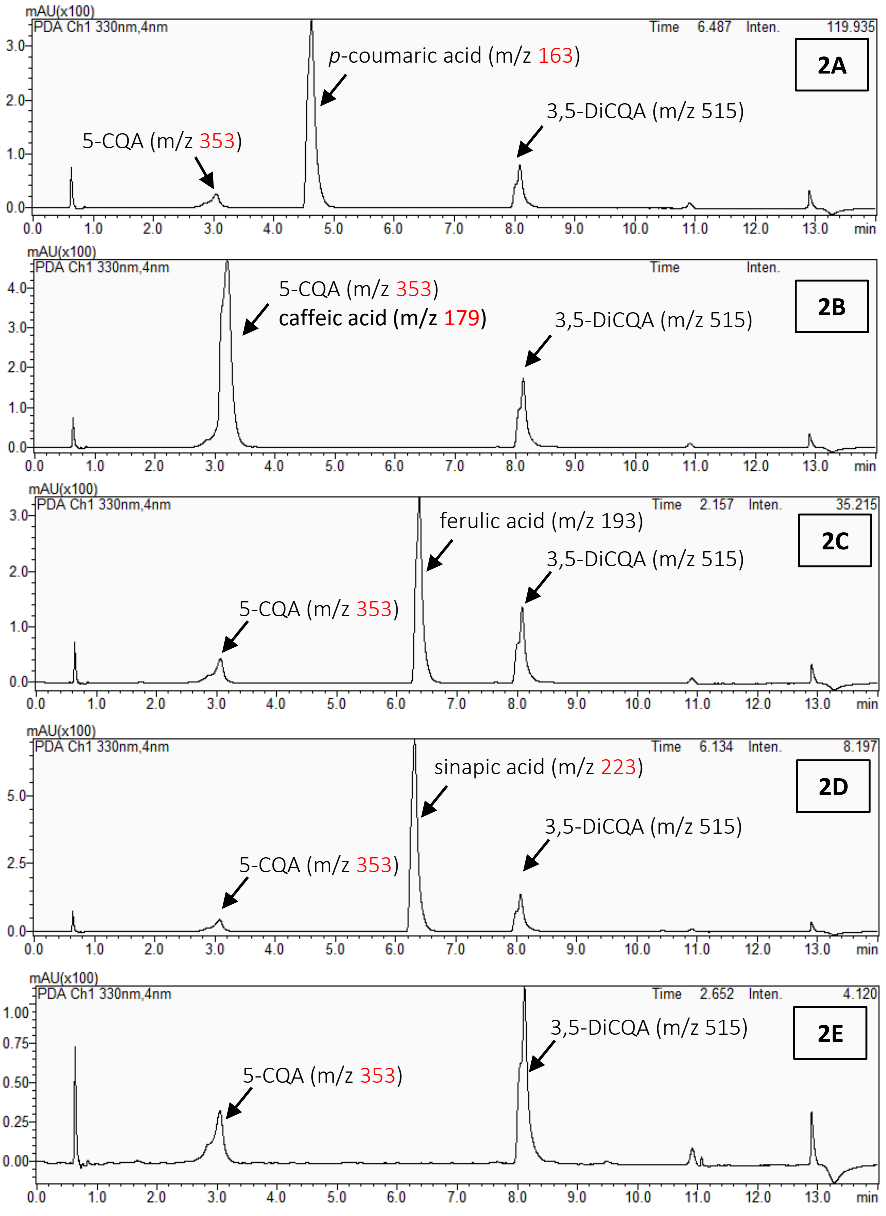

Supplementary Figure 10: Evaluation of IbICS activity with other hydroxycinnamate derivatives as acyl donors and quinic acid and 5-CQA as acyl acceptors.
Incubations were performed with 5 mM acyl donor *p*-coumaric acid (A), caffeic acid (B), ferulic acid (C) or sinapic acid (D), *trans*-cinnamic acid (E), and 5 mM acyl acceptor (quinic acid (1) and 5-CQA (2)) at 25°C overnight at pH 6. The activity of IbICS was confirmed in contact with 10 mM 5-CQA by the production of 3,5-diCQA and quinic acid (+).

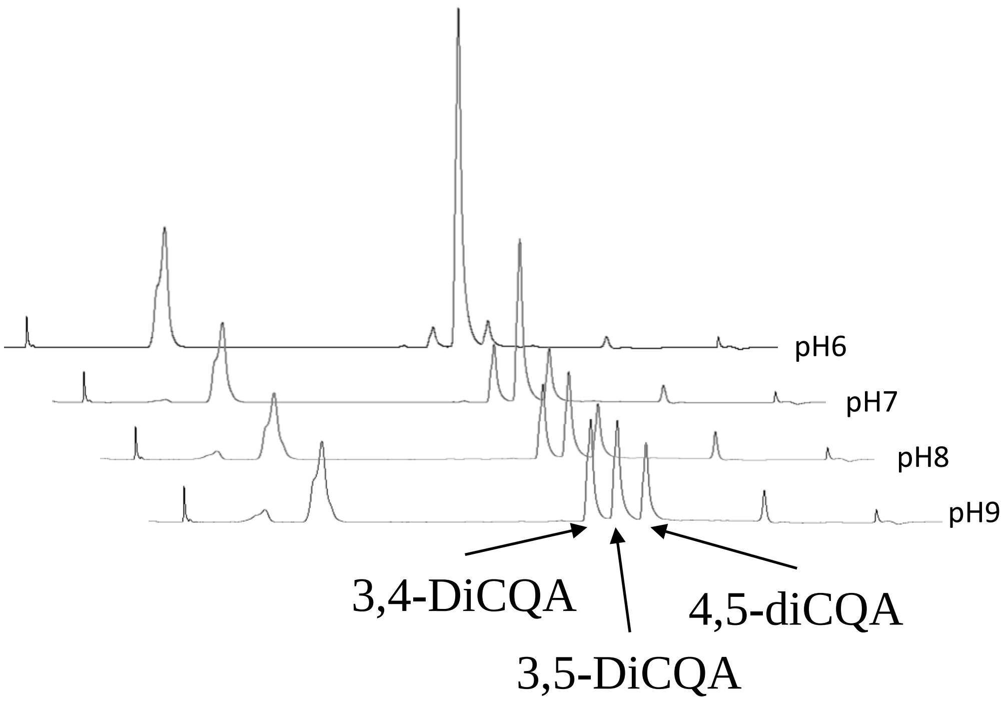

Supplementary Figure 11: Assessment of the stability of the chemical stability of 3,5-diCQA incubated during 15h at 30°C in buffers with different pH.

Accession Nr	Putative function	Acrylamide slice ID	Peptide Sequences
XM_019330300.1	Ipomoea nil 14-3-3 protein 1 (LOC109180616)	5, 6	AAQDIAAADLAPTHPIR, AAQDVAVADLAPTHPIR, AAQEVALADLSPTHPIR, AFDEAISELDTLNEESYK, AFDEAISELDTLNEESYKDSTLIMQLLR, DNLTLWTSDAQDQLDEP, DNLTLWTSDMQDDGTDEIK, DNLTLWTSDMQDDGTDEIKEAQNK, DNLTLWTSDTADDAGDEIK, AYEAATTTAEADLPPTHPIR, AAADGAEELTVEER, ACNLAKQAFDEAIAELDTLGEESYK, DSTLIMQLLRDNLTLWTSDMQDDGADEIK, DSTLIMQLLRDNLTLWTSDMQDDGTDEIK, DSTLIMQLLRDNLTLWTSDMQDDGTDEIKEAQNK, EAADDTMNSYK, EQSVYMAKLAEQAERYEEMVK, GDYHRYLAEFKVGDER, GDYYRYLAEFKIGNER, GILNLLDSTLIDSASSSDSK, LGLALNFSVFYYEILNASEKACGMAKQAFEEAIAELDTLGEESYK, LGLALNFSVFYYEILNSPDR, LLDQYLIPSASTSESK, LLESNLVPSASSSESK, LVLGSTPAAELTVEER, MAAALEGNLSREQYVYLAK, MAREENVYMAKLAEQAER, MWWDLGEHRGGSCHPLSK, QAFDEAIAELDTLGEESYK, QAFDEAIAELDTLGEESYKDSTLIMQLLR, QAFDEAIAELDTLGEESYKDSTLIMQLLRDNLTLWTSDMQDDGADEIK, QAFDEAIAELDTLGEESYKDSTLIMQLLRDNLTLWTSDMQDDGTDEIK, QAFDEAISELDTLGEESYK, QAFDEAISELDTLGEESYKDSTLIMQLLR, QAFDEAISELDTLGEESYKDSTLIMQLLRDNLTLWTSDTADDAGDEIK, QAFEEAIAELDTLGEESYK, RAFDEAISELDTLNEESYK, SAQDIALGELAPTHPIR, SAQEIANAELAPTHPIR, SKIEAELTSICKGILNLLDSTLIDSASSSDSK, TVDVDELTVEER, VETELTNICNDIMVLMDEHLIPSCTAGESNVFYYKMK, VETELTNICNDIMVLMDEHLIPSCTAGESNVFYYKMKGDYYR, VVAAADGAEELTVEER
XM_019320443.1	Ipomoea nil 14-3-3-like protein B (LOC109171366)	6	ACHLAKQAFDEAISELDNLNEESYK, AESELSPTHPIR, AESELSPTHPIRLGLALNFSVFYYEIMNSSEKACHLAK, AYQAATTKAESELSPTHPIRLGLALNFSVFYYEIMNSSEK, QAFDEAISELDNLNEESYKDSTLIMQLLR, QAFDEAISELDNLNEESYKDSTLIMQLLRDNLTLWTSDAPEDGEEGQK, YLAEFKAADERK
XM_019341199.1	Ipomoea nil heat shock cognate protein 80-like (LOC109190650)	4, 5, 6	ADLVNNLGTIAR, ANNTLTIIDSGIGMTK, AQALRDSSMAGYMSSK, DEYAAFYKSLTNDWEEHLAVK, ELISNASDALDK, HNDDEQYVWESQAGGSFTVTR, HSEFISYPISLWIEK, HSEFISYPISLWTEK, ITLYLKEDQLEYLEER, KPEEITKDEYAAFYK, KTMEINPENPIMEELR, LDAQPELFIHIIPDK, LDAQPELFIHIVPDK, MAAETETFAFQAEINQLLSLIINTFYSNK, TMEINPENPIMEELR, TNNTLTIIDSGVGMTK, VVDSPCCLVTGEYGWTANMER

Accession Nr	Putative function	Acrylamide slice ID	Peptide Sequences
XM_01931 4302.	Ipomoea nil phospholipase D alpha 1 (LOC109165505)	3, 4, 5, 6	AGLVSGKDNIIDR, AYIPVEELLEGEEVDK, AYIPVEELLEGEEVDKWVEILDK, AYIPVEELLEGEEVDKWVEILDKDK, CWEDVFDAITNAK, DDNPVGATLIGR, DGLMATHDEETEQFFKDTDVHCVLCPR, DSEIAMGAYQPYHLAGR, ELSEIIIPPSPVMFPDDQETWNVQLFR, FTVYIVVPMWPEGIPESASVQAILDWQRR, GGPREPWHDIHSR, IPLSGGQYYEPHR,>IVVVDHEMPSGGSEQR, IVVVDHEMPSGGSEQRR, KSGEYEPSETPEPDTDYMR, KVNDIADKYWDLYSSESLER, LEGPIAWDVLYNFEQR, LLEEPNNPQWNESFHIYCAHSASNVIFTVK, MAQFLLHGTLHVTIYEIDQLQGEGGGGGGGFFRKIMEHVEEAVHIGK, MMIVDDEYIIVGSANINQR, NPDDGGSFVQDLQISTMFTHHQK, PLSGGQYYEPHR, RPKPGGDITLGELLK, SGEYEPSETPEPDTDYMR, SIDGGAAFGFPDTPEGAAK, TLDTAHHDDFHQPNFPDASITK, TSVGLLKKDGLMATHDEETEQFFK, TYLTFFCVGNR, VCMLVWDDR, VEDIGALHLVAK, VGRTRLLEEPNNPQWNESFHIYCAHSASNVIFTVK, VNDIADKYWDLYSSESLER, VSLYQDAHIPDGFVPR, WVEILDKDKNPINEGSK, YPGVPYTFFGQR, YPIGISSDGDVTELPGFEFFPDTK, YWDLYSSESLER)
XM_01931 3071.1	Ipomoea nil GDSL esterase/lipase At1g28590-like (LOC109164567)	3, 4, 5	AIAQAVHELVELGAR, ELLDGSLFVLGEFGGNDYTHSLLSGK, GINVIRPFIPIVTR, LEPDGTVPHMYFPPYGETYFDKPTGR, LIVDLIAQHYGLPLPPPSIPASLEGDK, NVIRPFIPIVTR
XM_01931 0914.1	Ipomoea nil proteasome subunit alpha type-7 (LOC109162181)	6	AITVFSPDGHLFQVEYALEAVR, EFLEKNYKETSGQETVK, IVNLDDHIALACAGLK, KYTQSGGVRPFGLSTLIIGFDPYTGVPSLYQTDPSGTFSAWKANATGR, NYKETSGQETVKLAIR, QLEEAEIDAIVAEIEAEK, YTQSGGVRPFGLSTLIIGFDPYTGVPSLYQTDPSGTFSAWK
XM_01932 1217.1	Ipomoea nil mitogen-activated protein kinase homolog NTF4 (LOC109172058), transcript variant X1	3	ASFNDVYIAYELMDTDLHQIIR, FIQYNIFGNVFEVTAK
XM_01933 1679.1	Ipomoea nil pyruvate decarboxylase 1 (LOC109181768)	3, 4, 5, 6	AVKPVIIGGPK, LSNQMGLEAAVEAAAEFLNK, VGRTRLLEEPNNPQWNESFHIYCAHSASNVIFTVK, VISCIGDGSFQVTAQDVSTMLR, AYEAATATASTDLPPTHPIR,DNLTLWTSDLPEEGGEQSK, QAFDEAIAELDSLNEESYK, QAFDEAIAELDSLNEESYKDSTLIMQLLRDNLTLWTSDLPEEGGEQSK
XM_01930 3178.1	Ipomoea nil 14-3-3 protein 9-like (LOC109155578)	5, 6	AYETATTTAEAELPPTHPIR, AYETATTTAEAELPPTHPIRLGLALNFSVFYYEIMNSPER, DNLTLWTADIPEDAEDGQK, IAEQAERYDEMVDAMKK, LGLALNFSVFYYEIINSPERACHLAK, QAFDEAIAELDSLNEDSYK, QAFDEAIAELDSLNEDSYKDSTLIMQLLRDNLTLWTADIPEDAEDGQK)
AF109123.1	Ipomoea batatas anionic peroxidase Swpa3 (swpa3)	3, 5	CPLLCVPIFINPAR, EVVDAAIDAETR, FTGETYTVTLGR, GFTETELVALLGVHTVGFSR, YRDEMDAFLADFAAAMVK
M64362.1	Sweet potato starch phosphorylase	3, 4, 5	DELEEKDTELEK, DFPSYIECQEK, FADNEDLQIEWR, FAMNGCILIGTLDGANVEIRQEVGEENFFLFGAEAHEIAGLR, FITDVGATINHDPEIGDLLK, KFADNEDLQIEWR, MANLCVVGGHAVNGVAEIHSDIVK, QEVGEENFFLFGAEAHEIAGLR, TANLTGANTQLVGPNEELASQVEK, TGYSVSPNAMFDIQVK, WIGTEDWVLNTEK

Accession Nr	Putative function	Acrylamide slice ID	Peptide Sequences
AF259983.1	Ipomoea batatas putative cysteine protease	3, 4	DFQLYSSGIFTGSCGTDLDHGVTAVGYGTENGVDYWIVK, EAVAGQPVSVAIEAGGR, NGGIDTEADYPYTGR, NSWAASWGEK, VVSIDGYEDVTPYDEAALK
XM_019344884.1	Ipomoea nil luminal-binding protein 4 (LOC109194034)	3, 6	DILLLDVAPLTLGIETVGGVMTK, EPNKGVNPDEAVAFGAAVQGGILSGEGGDETK,FDLTGIAPAPR, ITPSWVGFTDNER, KSQVFTTYQDQQTTVTIQVFEGER, SQVFTTYQDQQTTVTIQVFEGER)
XM_019319061.1	Ipomoea nil annexin D1 (LOC109170102)	4	DLEADEKDEFLAILR, DTHGDYEDMLLALLGHGDI, QAYADTYGEDILK, SLEEDVAQHTTGDFR, SLEEDVAQHTTGDFRK
XM_019343001.1	Ipomoea nil heat shock protein 90-6, mitochondrial (LOC109192387)	4	DLEVLFLVDPIDEVAIQNLK, EIYYIASDSVASAK
XM_019297162.1	Ipomoea nil low-temperature-induced cysteine proteinase-like (LOC109149412)	3, 4, 5	DQGSCGSCWAFSTIAAVEGVNQLATGNLISLSEQELVDCDRK, FADITNEEYRTGYLGSKR, INQGCNGGDMDYAFQFIIK, NGGIDSEEDYPYTGKDGK, NSWGDYWGEK, SGLCGIAMEASYPTKTGDNPPPSPPSPPSPTPAPPSPSAPSVCDK, VASIDGYEDVPINNEK
XM_019297245.1	Ipomoea nil fructose-bisphosphate aldolase 6, cytosolic (LOC109149463)	5	ELLFTTPGALK, YLSGVILFEETLYQK
XM_019316225.1	Ipomoea nil aspartic proteinase-like (LOC109167232)	4, 5	FTVIFDTGSSNLWVPSSK, NYLDAQYYGEISIGSPPQK
XM_019311849.1	Ipomoea nil ribonuclease TUDOR 1-like (LOC109163119), transcript variant X2	5	GAVQSPTDEFEVFYIDYGNQEAVPYSK, IWANYVEGEEVPTGPVSERRQK, KGDIVLAQFSADNSWNR
XM_019331998.1	Ipomoea nil ferritin-3, chloroplastic-like (LOC109182001)	5	GDALYAMELALSLEK
XM_019305920.1	Ipomoea nil proteasome subunit alpha type-6-like (LOC109158098)	6	NDPAFSYEETVQTAISALQSVLQEDFK, NDPAFSYEETVQTAISALQSVLQEDFKASEIEVGVVRQEDPSFR, QEDPSFRVLSTEEIDEHLTAISER, SQVYTQHAYMRPLGVVAMVLGIDDEK, SQVYTQHAYMRPLGVVAMVLGIDEEK, VLSTEEIDEHLTAISER, VLSTEEIDEHLTAISERD, YGYEMPVDVLAR
XM_019298071.1	Ipomoea nil 2-Cys peroxiredoxin BAS1, chloroplastic (LOC109150163)	3	SYNVLIPDQGIALR, SYNVLIPDQGIALRGLFIIDK
XM_019339350.1	Ipomoea nil isovaleryl-CoA dehydrogenase, mitochondrial (LOC109188731)	3	VADMYTALQSSR
XM_019321471.1	Ipomoea nil luminal-binding protein 4-like (LOC109172297)	3, 6	VEIIANDQGNR, VQQLLKDYFDGK

Supplementary table 1: List of the different peptides obtained by LC MS/MS analysis and the corresponding proteins identified in public databases.
