# Supplementary material for: A GDSL lipase-like from Ipomoea batatas catalyzes efficient production of 3,5-diCQA when expressed in Pichia pastoris
Source: Commun Biol. 2020 Nov 13;3:673. doi: 10.1038/s42003-020-01387-1 (PMC7666189; doi:10.1038/s42003-020-01387-1)
Supplement: Supplementary file 4 — Supplementary Data 2 [file 42003_2020_1387_MOESM4_ESM.pdf]

Incubation :30min à 6°C à 42°C
Echantillons: GDSL purifiée avec un dosage de 0,410mg/ml (Bradford+qBit) - pureté de 5%
Extraction: Protéine dessalée et éluée dans PBSx1 ph 7.4

Incubation avec CGA à la concentration finale de 10mM
Utilisation d'un polytampon (0.1MTris, 20mM MES, 0.1M acide acétique) à ph6.5
Solution mère CGA 100mM dans Tampon PBSx1 + 1g/L MES, pH3
Réaction: 2µl echantillon protéique +5µl CGA 100mM + 43µl Tp ph6.5
Arrêt de la réaction avec 150µl d'EtOH absolu pour 50µl de réaction
Vortex pendant 30 secondes après incubation, centrifugation 10 minutes Vit max

Date manip n°1: 20190115

Data Filename	Ret. Time	Area	Teneur en 3,5-DCQ (mg/L)	Prise en compte de la dilution par 4	Moyenne (mg/L)	ET (mg/L)	Teneur en 3,5-DCQ (mM)	Moyenne (mmol/L)	ET (mM)	Bioconversion (%)	Moyenne (%)	ET(%)
GDSL_6°C_n°3.l	-----	0	0	0			0			0		
GDSL_6°C_n°2.l	-----	0	0	0			0			0		
GDSL_6°C_n°1.l	-----	0	0	0	0	0	0	0	0	0	0	0
GDSL_16°C_n°3	8.203	222672	14,117746	56,470982			0,3666947			5,092981788		
GDSL_16°C_n°2	8.190	301410	19,109855	76,439421			0,4963599			6,893887156		
GDSL_16°C_n°1	8.192	321986	20,414405	81,65762	71,52267	10,034462	0,5302443	0,4484695	0,065158841	7,364503998	6,450457647	0,904983906
GDSL_18°C_n°3	8.197	321656	20,393483	81,57393			0,5297008			7,356956196		
GDSL_18°C_n°2	8.205	360703	22,869122	91,476488			0,5940032			8,250044056		
GDSL_18°C_n°1	8.196	359273	22,778458	91,113832	88,05475	4,3205466	0,5916483	0,5606746	0,028055497	8,217336917	7,941445723	0,389659685
GDSL_20°C_n°3	8.227	406090	25,746727	102,98691			0,6687462			9,288141187		
GDSL_20°C_n°2	8.212	403128	25,558932	102,23573			0,6638684			9,220393954		
GDSL_20°C_n°1	8.208	410855	26,048836	104,19534	103,1393	0,7040106	0,6765931	0,6726696	0,004571497	9,397126862	9,301887334	0,063493019
GDSL_22°C_n°3	8.228	492014	31,19444	124,77776			0,8102452			11,25340564		
GDSL_22°C_n°2	8.218	456864	28,965877	115,86351			0,7523604			10,44945046		
GDSL_22°C_n°1	8.216	0	0	0	120,3206	4,4571276	0	0,7813028	0,028942387	0	10,85142805	0,401977595
GDSL_25°C_n°3	8.208	592853	37,587787	150,35115			0,9763062			13,55980784		
GDSL_25°C_n°2	8.218	596225	37,801577	151,20631			0,9818592			13,63693265		
GDSL_25°C_n°1	8.211	488993	31,002904	124,01162	141,8564	11,896494	0,8052702	0,8907882	0,077249961	11,18430896	12,79368315	1,072916129

GDSL_28°C_n°3	8.212	726927	46,088284	184,35314			1,1970983			16,62636511		
GDSL_28°C_n°2	8.224	600913	38,098804	152,39521			0,9895793			13,74415717		
GDSL_28°C_n°1	8.201	539354	34,195869	136,78348	157,8439	17,672796	0,8882044	1,0426513	0,114758416	12,33617204	14,23556477	1,593866894
GDSL_30°C_n°3	8.223	748651	47,465619	189,86248			1,2328732			17,12323915		
GDSL_30°C_n°2	8.206	643763	40,815559	163,26224			1,0601444			14,72422772		
GDSL_30°C_n°1	8.223	731046	46,349435	185,39774	179,5075	10,830165	1,2038814	1,2183773	0,070325746	16,7205754	16,18934742	0,976746468
GDSL_32°C_n°3	8.189	830843	52,676717	210,70687			1,3682264			19,00314484		
GDSL_32°C_n°2	8.206	967370	61,332738	245,33095			1,5930581			22,12580743		
GDSL_32°C_n°1	8.207	940361	59,620326	238,48131	231,5064	13,866337	1,5485799	1,4584032	0,090041151	21,50805421	20,87900216	1,250571547

25°C

GDSL_lot2_2µl.	0	0	0	0			0			0		
GDSL_lot2_5µl.	8.210	479754	30,417138	121,66855			0,7900555			10,97299339		
GDSL_lot2_7.5µ	8.209	1078528	68,380325	273,5213			1,7761124			24,66822709		
GDSL_lot2_10µ	8.224	1323463	83,909579	335,63832			2,1794696			30,27041099		
GDSL_lot2_20µ	8.208	2818852	178,71953	714,87812			4,6420657			64,47313493		

35DCQ_100mgL_1.lcd 0 8.183 1577249

Date manip n°2: 20190117

Data Filename	Ret. Time	Area	Teneur en 3,5-DCQ (mg/L)	Prise en compte de la dilution par 4	Moyenne (mg/L)	ET (mg/L)	Teneur en 3,5-DCQ (mM)	Moyenne (mmol/L)	ET (mM)	Bioconversion (%)	Moyenne (%)	ET(%)
GDSL_28°C_n°1	8.307	703261	45,647195	182,58878			1,1856414			16,46724208		
GDSL_28°C_n°2	8.271	699690	45,415409	181,66164			1,179621			16,38362516		
GDSL_28°C_n°3	8.297	536714	34,836979	139,34792	167,8661	19,01213	0,9048566	1,0900397	0,123455389	12,56745272	15,13943999	1,714658178
GDSL_30°C_n°1	8.264	810666	52,618628	210,47451			1,3667176			18,98218907		
GDSL_30°C_n°2	8.261	804704	52,231647	208,92659			1,3566662			18,84258557		
GDSL_30°C_n°3	8.266	814978	52,898511	211,59404	210,3317	0,9367508	1,3739873	1,3657904	0,006082798	19,08315692	18,96931052	0,084483299
GDSL_32°C_n°1	8.253	895454	58,122039	232,48816			1,5096634			20,9675466		

GDSL_32°C_n°2	8.238	851368	55,260509	221,04204			1,4353379			19,93524873		
GDSL_32°C_n°3	8.249	880579	57,156534	228,62614	227,3854	4,2289372	1,4845853	1,4765289	0,027460631	20,61924032	20,50734522	0,381397658
GDSL_34°C_n°1	8.235	1068339	69,343642	277,37457			1,8011335			25,01574371		
GDSL_34°C_n°2	8.259	933582	60,596848	242,38739			1,5739441			21,86033464		
GDSL_34°C_n°3	8.255	869071	56,409574	225,6383	248,4668	19,271877	1,4651837	1,6134205	0,125142058	20,34977419	22,40861751	1,738084134
GDSL_36°C_n°1	8.264	1016246	65,962394	263,84958			1,7133089			23,79595754		
GDSL_36°C_n°2	8.281	925834	60,093941	240,37576			1,5608816			21,67891097		
GDSL_36°C_n°3	8.227	998871	64,83462	259,33848	254,5213	9,4303391	1,6840161	1,6527355	0,061235968	23,38911239	22,9546603	0,850499554
GDSL_38°C_n°1	8.263	1086796	70,541647	282,16659			1,8322506			25,44792449		
GDSL_38°C_n°2	8.211	880523	57,152899	228,6116			1,4844909			20,61792905		
GDSL_38°C_n°3	8.261	1072512	69,614502	278,45801	263,0787	22,978089	1,8081689	1,7083034	0,149208371	25,1134568	23,72643678	2,072338487
GDSL_40°C_n°1	8.286	1006319	65,318054	261,27221			1,6965728			23,56351139		
GDSL_40°C_n°2	8.303	999653	64,885378	259,54151			1,6853345			23,40742335		
GDSL_40°C_n°3	8.290	980983	63,673547	254,69419	258,5026	2,5389663	1,6538584	1,6785886	0,016486794	22,97025505	23,31372993	0,228983251
GDSL_42°C_n°1	8.293	791858	51,39784	205,59136			1,3350088			18,54178944		
GDSL_42°C_n°2	8.292	937144	60,82805	243,3122			1,5799493			21,94374082		
GDSL_42°C_n°3	8.272	1101609	71,503127	286,01251	244,972	27,360324	1,8572241	1,5907274	0,177664444	25,79477901	22,09343642	2,467561726

35DCQ_100mgl	8.256	1583857	1540644,5
35DCQ_100mgl	8.252	1497432	

CGA (mM)	3,5-DCQ (m	Acide quinique (mM)
10	7,2	2,8

Teneur en 3,5-DCQ (mmol/L)			EC (mM)			Bioconversion (%)			EC (%)		
20190115	20190117	Moyenne	20190115	20190117	Moyenne	20190115	20190117	Moyenne	20190115	20190117	Moyenne

6°C	0		0	0		0	0		0	0		0
16°C	0,4484695		0,4484695	0,0651588		0,0651588	6,4504576		6,450457647	0,904983906		0,904983906
18°C	0,5606746		0,5606746	0,0280555		0,0280555	7,9414457		7,941445723	0,389659685		0,389659685
20°C	0,6726696		0,6726696	0,0045715		0,0045715	9,3018873		9,301887334	0,063493019		0,063493019
22°C	0,7813028		0,7813028	0,0289424		0,0289424	10,851428		10,85142805	0,401977595		0,401977595
25°C	0,8907882		0,8907882	0,07725		0,07725	12,793683		12,79368315	1,072916129		1,072916129
28°C	1,0426513	1,0900397	1,0663455	0,1147584	0,123455	0,1191069	14,235565	15,13944	14,68750238	1,593866894	1,714658178	1,654262536
30°C	1,2183773	1,3657904	1,2920838	0,0703257	0,006083	0,0382043	16,189347	18,969311	17,57932897	0,976746468	0,084483299	0,530614884
32°C	1,4584032	1,4765289	1,467466	0,0900412	0,027461	0,0587509	20,879002	20,507345	20,69317369	1,250571547	0,381397658	0,815984603
34°C		1,6134205	1,6134205		0,125142	0,1251421		22,408618	22,40861751		1,738084134	1,738084134
36°C		1,6527355	1,6527355		0,061236	0,061236		22,95466	22,9546603		0,850499554	0,850499554
38°C		1,7083034	1,7083034		0,149208	0,1492084		23,726437	23,72643678		2,072338487	2,072338487
40°C		1,6785886	1,6785886		0,016487	0,0164868		23,31373	23,31372993		0,228983251	0,228983251
42°C		1,5907274	1,5907274		0,177664	0,1776644		22,093436	22,09343642		2,467561726	2,467561726
